# A psychometric evaluation of the German Revised-Green et al. Paranoid Thoughts Scale (R-GPTS) in clinical and non-clinical groups

**DOI:** 10.1186/s12888-025-07538-0

**Published:** 2025-11-18

**Authors:** Stephanie Rek, Matthias A. Reinhard, Daniel Freeman, Peter Falkai, Frank Padberg

**Affiliations:** 1 Department of Psychiatry and Psychotherapy, LMU University Hospital Munich, Munich, Germany; 2 Department of Experimental Psychology, University of Oxford, Oxford, UK; 3 Max Planck Institute of Psychiatry, Munich, Germany; 4 German Center for Mental Health (DZPG), Partner Site Munich-Augsburg, Munich, Germany

**Keywords:** Paranoia, R-GPTS, Psychometric evaluation, Measurement invariance

## Abstract

**Background:**

Paranoia, characterised by inaccurate fears that others intend to cause harm, can significantly affect social functioning. Research has demonstrated that paranoia exists on a spectrum of severity, with milder forms prevalent in the general population. The Revised-Green et al. Paranoid Thoughts Scale (R-GPTS) is the most commonly used measure of paranoia comprising a scale to assess ideas of reference and a scale to assess ideas of persecution. The aim of the study was to validate a German version of the R-GPTS and assess its psychometric properties in non-clinical and clinical groups.

**Methods:**

This longitudinal study was conducted in Germany, including a non-clinical group (*n* = 601) recruited online and a clinical group of inpatients diagnosed with persistent depressive disorder (*n* = 102). Participants completed an online survey assessing paranoia, other psychotic experiences, depression, and anxiety. Statistical analyses included confirmatory factor analysis to evaluate the factor structure and measurement invariance across sex, time, and patient status. McDonald’s omega was estimated for internal consistency, and Spearman correlations for test-retest reliability, and convergent and discriminant validity.

**Results:**

Confirmatory factor analysis supported the two-factor structure, solid evidence in favour of metric invariance for the R-GPTS A subscale and more mixed measurement invariance evidence for the R-GPTS B subscale. The German R-GPTS exhibited good-to-excellent internal consistency (McDonald’s omega : 0.87 to 0.92), test-retest reliability analyses showed moderate-to-strong stability over a 10-week period, and we observed evidence for convergent and discriminant validity.

**Discussion:**

These findings suggest that the German version of the R-GPTS is a reliable and valid tool for assessing paranoid thoughts across various populations. However, caution is warranted when interpreting score differences, as measurement non-invariance may impact the comparability of results for the Persecutory Ideations subscale. Limitations include potential selection bias in the non-clinical group and a focus solely on persistent depressive disorder in the clinical group.

**Conclusions:**

This study confirms the psychometric robustness of the German R-GPTS and contributes to the understanding of paranoia assessment in diverse populations, highlighting the need for further research to explore its applicability across different psychiatric conditions.

**Supplementary Information:**

The online version contains supplementary material available at 10.1186/s12888-025-07538-0.

## Introduction

By nature, humans have always been social animals trying to ensure survival. For many people, social interactions are perceived as satisfactory and supportive, but they can also be a source of difficulties. Adaptation to the social world is often particularly challenging for individuals with a diagnosis of a mental health disorder who can often present with difficulties trusting others [1]. Paranoia has been defined as “the unfounded fear that others intend to cause you harm” [2,3] and is an excessive form of mistrust. Traditionally, paranoia has been studied in its severest form (i.e. persecutory delusions) in psychotic disorders such as schizophrenia (e.g., 4]). However, research has accumulated showing that paranoia is best represented on a spectrum of severity in the general population. Many individuals experience a few paranoid thoughts and a few experience many paranoid thoughts [5, 6]. Descriptively, it has been estimated that about one quarter of the general population report being mistrustful of other people [7]. These milder forms of paranoia have been associated with multiple adverse health outcomes (e.g., anxiety, depression, and insomnia; [8,9]), that are presumed to be more severe for increasing levels of paranoia including in various mental disorders. To make conclusive statements on specific risk levels posed by varying levels of paranoia, however, paranoia needs to be assessed validly and reliably across clinical and non-clinical groups.

The most used standard assessment tool for measuring paranoia on a spectrum of severity is the Green et al. Paranoid Thoughts Scale [GPTS; [10]]. This self-report measure was developed using data from individuals without a history of mental illness and patients with current persecutory delusions in the context of a psychotic disorder. It consists of two scales with 16 items each assessing “Part A: ideas of reference and other social-evaluative concerns” (e.g., ‘People definitely laughed at me behind my back’) and “Part B: persecutory ideations” (e.g., ‘People have intended me harm’). Scale development followed the theoretical foundation that ideas of reference and other social-evaluative concerns often precede but also form the basis of the formation of persecutory ideation [11]. A recent literature review on different assessment tools of paranoia recommended the GPTS as the best current measure of the construct. Yet, the authors of the review concluded that some psychometric properties required re-evaluation using larger samples [12].

In 2019, such a large-scale psychometric evaluation of the GPTS was conducted using an overall pooled sample of 10,551 individuals including from studies of patients diagnosed with psychosis and non-clinical paranoia in the general population [13]. Resulting from a problematic factor structure in the original GPTS Part A and local item dependencies in Part B, some items of the original scale were excluded leading to the introduction of a Revised-GPTS (R-GPTS) version. The R-GPTS comprises eight items assessing ideas of reference and 10 items measuring persecutory ideations, which provided a clean two-factor structure and excellent psychometric properties with high item discrimination, good item difficulty, high reliability (*α* values above 0.90), and measurement invariance for age and sex. Notably, the validation included latent construct ranges, establishing meaningful class categories from ‘average’ to ‘very severe’ levels of paranoia, with specific cut-offs for clinical (sum-score ≥ 11) and likely persecutory delusions (sum-score ≥ 18). These cut-offs correspond to optimal points for distinguishing between clinical patients and non-clinical individuals while minimizing false positives. Psychometric invariance was also established in individuals self-reporting psychiatric diagnoses versus those not self-reporting a diagnosis in 4 Polish samples spanning 2,129 individuals [14]. However, psychometric invariance over time and patient status in clinically ascertained psychiatric patients without primary presentation involving paranoia has not yet been evaluated.

The present study therefore translated the R-GPTS into the German language and aimed at replicating the two-factor structure, high observed internal consistency, and measurement invariance for sex. Moreover, we aimed at extending previous research with regards to test-retest reliability (over 10-weeks), convergent and discriminant validity using the short Community Assessment of Psychic Experiences-Positive Scale [CAPE‐P15; [15, 16]] and Depression, Anxiety and Stress Scales-21 [DASS-21; [17, 18]], and measurement invariance to ensure that the same underlying construct is being assessed across patient status and time (over 10-weeks).

## Method

### Study population

The present study testing the properties of a German version of the R-GPTS is based on a non-clinical group from the German general population and a clinical inpatient group of psychiatric patients with persistent depression disorder (PDD) from the psychiatric clinic of the Ludwig-Maximilians-University (LMU).

#### Non-clinical group

The non-clinical group was recruited using the software LimeSurvey between April 2020 and August 2021 via social media and university mailing lists for participation in a secure online survey. Participants needed to be aged 18 or older. There were no other exclusion criteria. To reward participation, participants received the chance to several 50 Euro vouchers. During the recruitment period, 681 individuals completed the online survey at baseline. To assure validity of responses, three bogus items were included in the survey (e.g., “Please indicate *completely agree*”) and 59 participants failing to answer > 1 bogus item correctly were excluded. Survey time of < 25 min was deemed unrealistic (Median response time: 48 min), which led to the exclusion of 8 additional participants. Finally, we used the *careless* package [version 1.2.2;, [19]] in *R* software [version 4.3.2;, [20]] to identify and exclude 13 careless responders that had the longest or average length of identical consecutive responses ≥ 3 standard deviations (SD) longer than average. This led to a final general population sample of 601 participants. 396 participants also fulfilled quality criteria at a 10-week follow-up and were considered for test-retest reliability and temporal invariance analyses (see below).

#### Clinical group

The clinical group was comprised of inpatients suffering from PDD who enrolled in a larger naturalistic 10-week treatment programme with Cognitive Behavioural Analysis System of Psychotherapy (CBASP). Inclusion criteria were age 18–65 years, fluency in German language and PDD diagnosis as assessed using a German version of the Structural Clinical Interview for DSM-IV or DSM-5 [21]. Patients were excluded if they displayed acute suicidality, had a diagnosis of bipolar disorder or psychosis, were pregnant, or if they presented with an unstable somatic condition requiring treatment. For the current study, a subset of 102 patients with available baseline data on the R-GPTS was included (since the questionnaire was introduced to the questionnaire battery later). For test-retest reliability and temporal invariance analyses (see below), we used data from the 10-week post-treatment assessment.

#### Group matching

To maximise comparability of clinical and non-clinical samples, a matched non-clinical subsample was identified from the full non-clinical sample using a matching procedure. Specifically, we used the *R* software package *MatchIt* [version 4.5.5;, 22]] to match non-clinical participants based on age and sex to the clinical sample. We tried to maximise the matching ratio of non-clinical participants to patients by identifying the highest possible ratio without significant differences in age or sex.

### Questionnaires

#### Paranoia

Ideas of reference and ideas of persecution over the past month were assessed with the German version 18-item Revised-Green et al. Paranoid Thoughts Scale [R-GPTS; [13]]. Items are rated on a five-point Likert scale ranging from 0 (not at all) to 4 (totally). Scores can range from 0 to 32 for the “Social Reference” (R-GPTS A) subscale and from 0 to 40 for the “Persecutory Ideations” (R-GPTS B) subscale, where higher scores indicate higher levels of reference and paranoia, respectively. Excellent psychometric properties of the scales have been reported [13]. The German version was translated from the original English version following common guidelines for forward and backward translation [23].

#### Depression and anxiety

The German version of the Depression, Anxiety and Stress Scales-21 [DASS-21; 17, 18] was used to measure depression and anxiety during the preceding week. Items are rated on a Likert scale of zero (did not apply to me at all) to three (applied to me very much or most of the time). For depression and anxiety, scores can each range from 0 to 21. Higher scores indicate greater levels of depression and anxiety. In clinical and non-clinical samples good psychometric properties of the scales have been reported [24].

#### Psychotic-like experiences

Lifetime frequency of persecutory ideation (seven items), bizarre experiences (seven items) and perceptual abnormalities (three items) were assessed with subscales of the German version of the 15-item Community Assessment of Psychic Experiences-Positive Scale [CAPE-P15; [15, 16]]. Items are rated on a four-point Likert scale ranging from 0 (never) to 3 (nearly always). Scores can range from 0 to 45, with higher scores indicating greater levels of positive psychotic-like experiences. Good psychometric properties of the scale have been reported in previous research (e.g., [15, 25]).

### Statistical analyses

All analyses were conducted using *R* software (version 4.3.2; [19]). For full transparency of procedures, scripts are provided on the Open Science Framework under https://osf.io/aue8c/.

#### Factor structure and measurement invariance

Factor structure of the R-GPTS was evaluated for the two R-GPTS subscales with the *lavaan* package (version 0.6–17; [26]) in the non-clinical group at baseline using confirmatory factor analysis (CFA). To this end, mean and variance-adjusted weighted least squares estimation was selected to account for ordinal item response scales and item non-normality (i.e., lavaan estimator: WLSMV). Model fit was evaluated using the Comparative Fit Index (CFI), the Tucker–Lewis index (TLI), the root mean square error of approximation (RMSEA), and the standardized root mean square residual (SRMR). CFI values above 0.95, TLI values above 0.95, RMSEA values below 0.06, and SRMR values below 0.08 were considered as acceptable fit similar to previous work [27].

In turn, measurement invariance was assessed across sex, time, and patient status using multi-group and longitudinal CFAs with increasingly restrictive levels of invariance [28, 29]. Specifically, we sequentially tested for configural invariance (testing whether the number of factors and pattern of factor loadings is the same across groups or time), metric invariance (additionally testing whether factor loadings are the same across groups or time), scalar invariance (additionally testing whether the item intercepts are the same across groups or time), and residual invariance (additionally testing whether item residuals are the same across groups or time). In addition to evaluating the same fit metrics as for the main CFA, we also tested for significant differences between fit of different invariance levels (i.e., configural vs. metric invariance, metric vs. scalar invariance, and scalar vs. residual invariance) using log-likelihood tests and considered significant differences as evidence of violation of invariance assumptions of the more restrictive model [28]. Once there was sufficient evidence against more restrictive models from log-likelihood tests, we stopped the sequence of invariance testing for the respective R-GPTS subscale and invariance grouping.

Of note, both CFA and measurement invariance analyses were conducted separately for respective R-GPTS subscales rather than in joint models. Separation of analyses for the subscales was selected based on evidence in favour of unidimensionality of subscale constructs observed by Freeman *and colleagues* [13]. This also allowed for separate model fit criteria estimates, so offered more nuanced interpretation of R-GPTS subscales.

#### Internal reliability

Internal reliability was evaluated using McDonald’s omega total. Omega total is based on fewer assumptions than Cronbach’s alpha and has been shown to be less sensitive to violation of assumptions than Cronbach’s alpha [30]. We considered values between 0.70 and 0.80 as acceptable, between 0.80 and 0.90 as good and above 0.90 as excellent.

#### Test-retest reliability

Test-retest reliability of the R-GPTS was evaluated using Spearman correlations and intra-class correlation coefficients (ICCs) of baseline and follow-up data in both non-clinical and clinical groups. ICC values of > 0.75 were considered excellent, between 0.60 and 0.75 as good, between 0.40 and 0.60 as fair and < 0.40 as poor.

#### Convergent & discriminant validity

Convergent validity was assessed by calculating Spearman’s rho correlation coefficients between R-GPTS subscale scores with CAPE-P15 subscales persecutory ideation, bizarre experiences and DASS-21 depression and anxiety scales. In turn, discriminant validity was tested by assessing significant differences in correlations between R-GPTS total and subscales with CAPE-P15 persecutory ideations as compared to correlations of respective R-GPTS scales with other CAPE-P15 and DASS-21 subscales. Here, the Steiger test was used to test for differences between the dependent correlations [31].

## Results

### Group characteristics

The characteristics of the non-clinical and clinical groups, including the subgroups for longitudinal analysis and matched patient versus control analyses, are summarised in Table 1. Briefly, the non-clinical sample included predominantly women (79%) and participants were relatively young (mean 30 years) while the clinical sample only included a slight majority of women (57%) and was substantially older (mean 40 years). Additionally, the non-clinical sample had an overall higher education level as reflected, for instance, by the greater completion rate of German A-levels (83%) compared to the clinical sample (43%).

**Table 1:** Baseline characteristics

	Non-clinical sample			Patients with PDD	
Variables	Full sample	With follow-up data	Matched sample	Full sample	With follow-up data
**n**	601	396	204	102	73
**Sex** (%)					
diverse	2 (0.3)	2 (0.5)	0 (0.0)	0 (0.0)	0 (0.0)
female	475 (79.0)	312 (78.8)	138 (67.6)	58 (56.9)	44 (60.3)
male	124 (20.6)	82 (20.7)	66 (32.4)	44 (43.1)	29 (39.7)
**School education (%)**					
Primary School	0 (0.0)	0 (0.0)	0 (0.0)	1 (1.0)	0 (0.0)
Intermediate School	15 (2.5)	7 (1.8)	10 (4.9)	13 (12.7)	12 (16.4)
Secondary School	54 (9.0)	33 (8.3)	31 (15.2)	24 (23.5)	17 (23.3)
Vocational Qualification	33 (5.5)	23 (5.8)	18 (8.8)	20 (19.6)	12 (16.4)
A-levels	499 (83.0)	333 (84.1)	145 (71.1)	44 (43.1)	32 (43.8)
**Academic education (%)**					
Never studied	86 (14.3)	52 (13.1)	48 (23.5)	43 (44.8)	32 (47.1)
Cancelled studies	35 (5.8)	24 (6.1)	23 (11.3)	16 (16.7)	9 (13.2)
Studying	299 (49.8)	197 (49.7)	47 (23.0)	4 (4.2)	3 (4.4)
Graduated	181 (30.1)	123 (31.1)	86 (42.2)	33 (34.4)	24 (35.3)
**Age** (mean (SD))	30.76 (11.56)	31.32 (11.89)	40.49 (13.08)	40.24 (12.92)	41.99 (12.71)
**R-GPTS A** (mean (SD))	7.31 (6.92)	6.86 (6.40)	6.40 (6.35)	8.00 (7.49)	7.85 (7.27)
**R-GPTS B** (mean (SD))	3.32 (6.22)	2.64 (4.87)	3.71 (6.41)	4.29 (7.08)	3.78 (6.03)
**R-GPTS A** (%)					
Average	418 (69.6)	288 (72.7)	151 (74.0)	65 (63.7)	47 (64.4)
Elevated	88 (14.6)	52 (13.1)	29 (14.2)	17 (16.7)	13 (17.8)
Moderately severe	60 (10.0)	39 (9.8)	15 (7.4)	13 (12.7)	9 (12.3)
Severe	22 (3.7)	13 (3.3)	5 (2.5)	6 (5.9)	3 (4.1)
Very severe	13 (2.2)	4 (1.0)	4 (2.0)	1 (1.0)	1 (1.4)
**R-GPTS B** (%)					
Average	466 (77.5)	320 (80.8)	159 (77.9)	76 (74.5)	56 (76.7)
Elevated	75 (12.5)	49 (12.4)	20 (9.8))	9 (8.8)	6 (8.2)
Moderately severe	31 (5.2)	17 (4.3)	13 (6.4)	10 (9.8)	7 (9.6)
Severe	23 (3.8)	10 (2.5)	10 (4.9)	6 (5.9)	4 (5.5)
Very severe	6 (1.0)	0 (0.0)	2 (1.0)	1 (1.0)	0 (0.0)

Note: ^a^School education groups follow the German school system with increasing education for Primary School (“Grundschule”), Intermediate School (“Hauptschule”), Secondary School (“Realschule”), Vocational Qualification (“Fachabitur”), and A-levels (“Abitur”)

The matching procedure resulted in a maximum matching ratio of 2:1 non-clinical to clinical individuals without significant differences in age (*p* = 0.874) or sex (*p* = 0.084) between the samples as assessed using Welch t-test and χ2-difference test, respectively. Matched non-clinical participants included 68% women and were 40 years old on average.

### Descriptive statistics

**Fig. 1:**
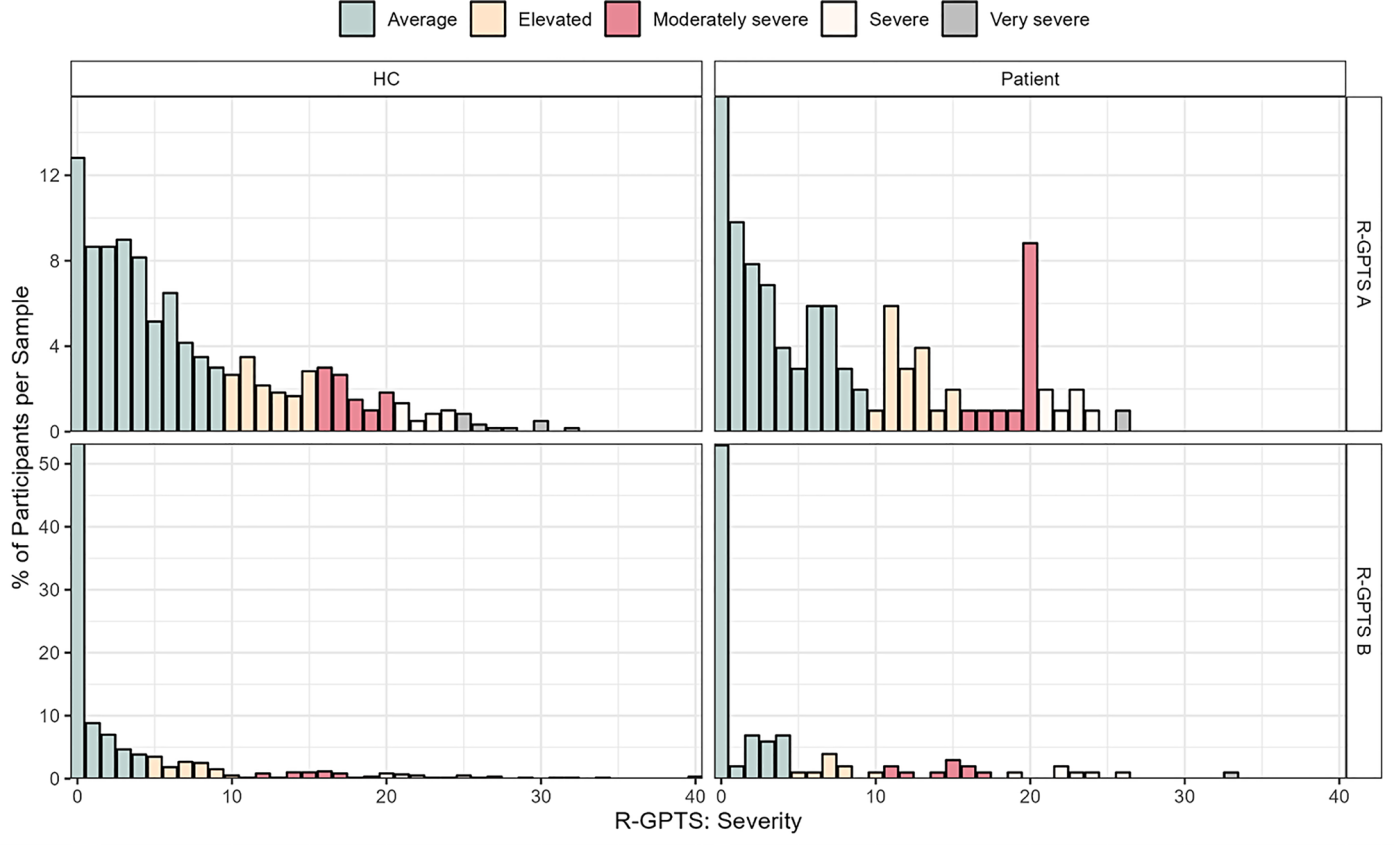
Bar chart of R-GPTS subscale distribution across samples. Note: Bar chart shows percentages of participants per sample for the full non-clinical and clinical samples, respectively

Item statistics and item intercorrelations for the non-clinical sample are provided in Supplementary Tables 1 and Supplementary Fig. 1, respectively, and score distributions for the R-GPTS in the full non-clinical and clinical samples at baseline are displayed in Fig. 1. Overall, R-GPTS items were right-skewed towards lower Likert responses with larger right skew for R-GPTS B than R-GPTS A items. This right skew translates directly to R-GPTS scale scores with 70% and 64% of participants in non-clinical and clinical groups, respectively, qualifying for the “average” category for R-GPTS A and 78% and 75% for R-GPTS-B (cf. Table 1). Item intercorrelations in the non-clinical sample showed correlations between 0.38 and 0.76 among R-GPTS A items, between 0.54 and 0.85 among R-GPTS B items, and between 0.34 and 60 among inter-subscale item combinations.

### CFA and measurement invariance

Fit metrics for CFA and measurement invariance models are displayed in Table 2.

**Table 2:** Confirmatory factor analysis (CFA) and measurement invariance test results

	*R*-GPTS A: Social Reference								*R*-GPTS B: Persecutory Ideations					
Analysis	χ^2^	SRMR	RMSEA		CFI	TLI		P-value^a^	χ^2^	SRMR	RMSEA	CFI	TLI	P-value^a^
CFA	42.967	0.041	0.044		0.997	0.996		-	53.42	0.050	0.030	0.999	0.999	-
**Gender invariance**														
Configural	92.059	0.033	0.066		0.973	0.962		-	481.230	0.063	0.140	0.882	0.849	-
Metric	95.106	0.036	0.058		0.975	0.97		0.945	535.009	0.082	0.139	0.87	0.851	0.105
Scalar	141.228	0.046	0.073		0.955	0.953		< 0.001**	555.698	0.083	0.133	0.866	0.863	0.003*
Residual	-	-	-		-	-		-	-	-	-	-	-	-
**Temporal invariance**														
Configural	249.820	0.045	0.064		0.948	0.935		-	813.174	0.076	0.102	0.855	0.826	-
Metric	256.244	0.048	0.061		0.949	0.940		0.825	852.505	0.089	0.101	0.848	0.829	0.414
Scalar	281.436	0.052	0.062		0.943	0.938		0.001*	865.074	0.09	0.098	0.848	0.838	0.224
Residual	-	-	-		-	-		-	953.926	0.092	0.101	0.830	0.829	0.237
**Patient vs. healthy control invariance**														
Configural	77.909	0.043	0.079		0.966	0.953		-	315.997	0.069	0.152	0.863	0.824	-
Metric	93.809	0.068	0.081		0.958	0.95		0.097	366.379	0.102	0.154	0.840	0.818	0.022*
Scalar	122.131	0.078	0.091		0.939	0.937		< 0.001**	-	-	-	-	-	-
Residual	-	-	-		-	-		-	-	-	-	-	-	-
**Patient Temporal invariance**														
Configural	186.42	0.093	0.115		0.879	0.847		-	689.559	0.121	0.214	0.619	0.544	-
Metric	225.14	0.189	0.127		0.838	0.811		0.004*	720.499	0.150	0.211	0.604	0.554	0.553
Scalar	-	-	-		-	-		-	734.270	0.156	0.206	0.601	0.576	0.136
Residual	-	-	-		-	-		-	1008.121	0.252	0.244	0.411	0.408	< 0.001**

Note: ^a^P-values are based on χ^2^-difference tests of more restrictive invariance models against less restrictive invariance models (i.e., metric invariance p-values are based on a test of metric vs. configural models, scalar invariance p-values are based on a test of scalar vs. metric models, and residual invariance p-values are based on a test of residual vs. scalar models). Once χ^2^-difference tests provided evidence against more restrictive invariance models, we stopped the invariance testing sequence thereafter resulting in empty rows. *P < 0.05. **P < 0.001

CFA of the R-GPTS subscales in the non-clinical group at baseline revealed acceptable fit on all fit metrics (i.e., SRMR, RMSEA, CFI, and TLI), thus confirming the validity of the suggested two R-GPTS subscale factors “Social Reference” and “Persecutory Ideations”. Additionally, evidence from factor loadings and cross-factor correlation analyses included in Supplementary Table 2 indicates clear separation of item associations to their respective factor.

Next, measurement invariance was tested across sex in the non-clinical group, across patient status by comparing clinical and non-clinical groups and across time in both non-clinical and clinical groups. Regarding sex, log-likelihood tests of measurement invariance models indicated significantly worse model fit for the scalar invariance models for both R-GPTS subscales (compared against metric invariance), but not yet with the metric invariance model (compared against configural invariance). This supports equal number of factors and factor loadings across groups. Regarding temporal invariance comparisons, evidence for the different measurement invariance thresholds differed between R-GPTS subscales and between analyses in clinical and non-clinical groups. Specifically, for R-GPTS A, evidence from log-likelihood tests supported metric invariance in the non-clinical group, but not in the clinical group. For R-GPTS B, evidence from log-likelihood comparisons supported residual invariance for R-GPTS B, but only scalar invariance in the clinical group.

In contrast to evidence from log-likelihood comparisons for sex and temporal invariance tests, however, fit measures were somewhat worse than for the main CFA. For R-GPTS A invariance models, SRMR values as well as most CFI and TLI values supported invariance models across sex while only SRMR values supported invariance over time in the non-clinical group. For patient status, there was consistent support from SRMR, CFI and TLI values for metric invariance. Temporal invariance in the non-clinical group showed worse fit measures than the CFA, which were substantially below the thresholds. For R-GPTS B, only SRMR values supported configural invariance models for sex and over time in the clinical group while none of the other fit measures provided evidence for any of the other models.

Taking both log-likelihood tests and fit measures together, there was solid evidence for metric invariance in R-GPTS A across sex and over time in the non-clinical sample as well as for patient status. For R-GPTS B, evidence was more mixed and only provided support for metric invariance across sex as well as scalar and residual invariance over time in non-clinical and clinical groups, respectively.

### Internal reliability

Internal reliability of the R-GPTS subscales was good-to-excellent with McDonald’s Omega values of 0.87 for R-GPTS A and 0.92 for R-GPTS B subscales.

### Test-retest reliability

Test-retest reliability analyses across 10-week follow-up time-points showed moderate-to-strong correlations of R-GPTS subscale scores in both the non-clinical (Spearman’s rho: 0.56–0.59) and clinical groups (Spearman’s rho: 0.51–0.63). In the non-clinical group, ICC values were good for the R-GPTS A subscale and fair for the R-GPTS B subscale. In the clinical group, the R-GPTS A ICC values were poor and fair for R-GPTS B, but test-retest reliability may have been affected by potential treatment effects (see Supplementary Table 3).

### Convergent and discriminant validity

**Fig. 2:**
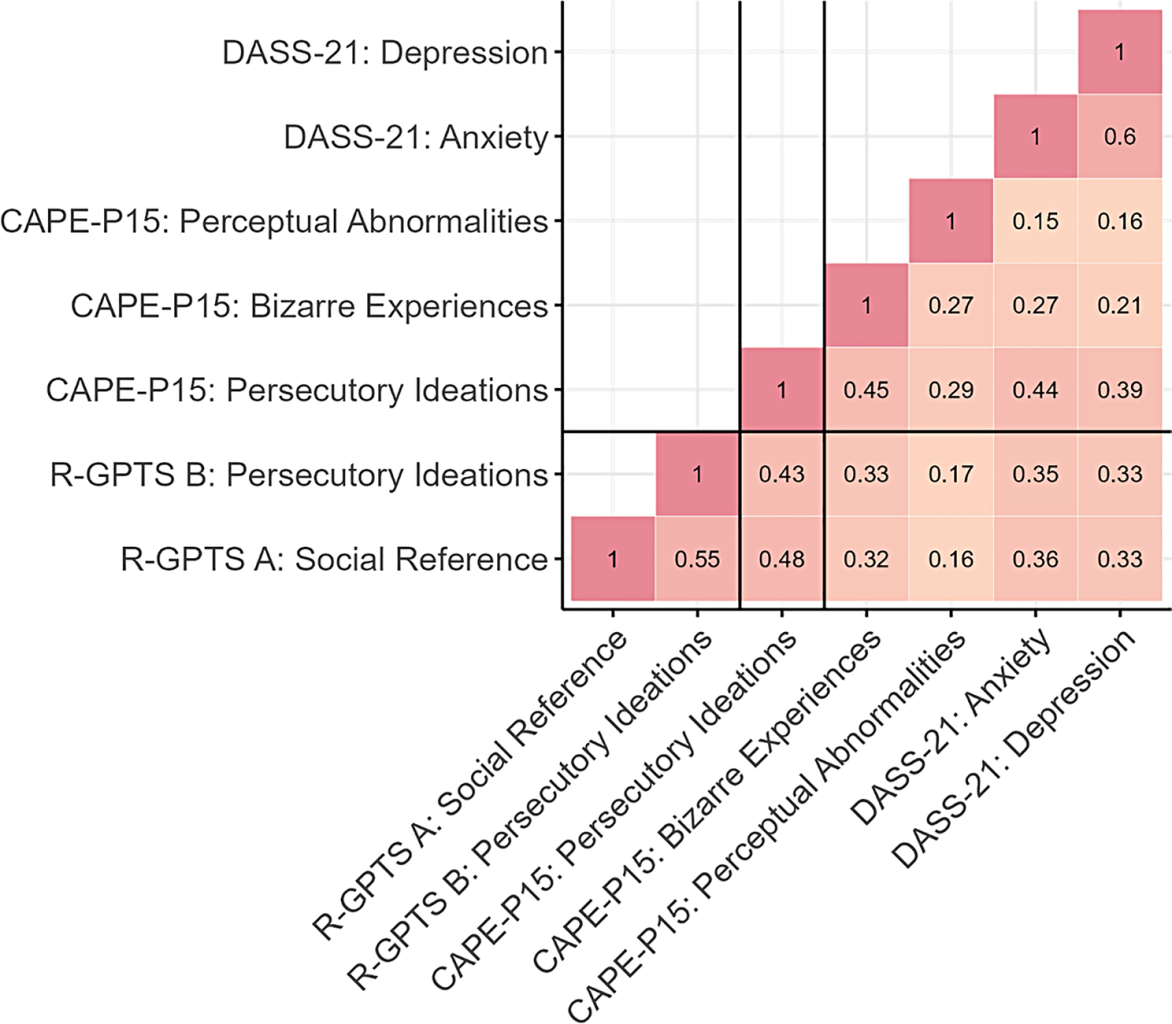
Correlation matrix of R-GPTS subscale scores with CAPE-P15 and DASS-21 scales

Correlation analyses showed evidence for convergent validity in that R-GPTS subscale scores exhibited significant moderate correlations to CAPE-P15 Persecutory Ideations in the non-clinical group with Spearman’s rho between 0.43 and 0.48 (see Fig. 2). Next, we used Steiger tests to compare these correlations to correlations with other CAPE subscales (Bizarre Experiences & Perceptual Abnormalities) and DASS Depression and Anxiety subscales to assess discriminant validity to other psychometric and psychopathological constructs. These results provided clear evidence for differences in correlation with CAPE Persecutory Ideations compared to other scales supporting discriminant validity (see Table 3).

**Table 3:** Convergent & discriminant validity

		Correlation Difference *P*-values^a^			
R-GPTS Subscale	Correlation with CAPE Paranoia	CAPE Bizarre	CAPE Abnormalities	DASS Depression	DASS Anxiety
A	0.48	< 0.001**	< 0.001**	< 0.001**	0.002*
B	0.43	0.008*	< 0.001**	0.008*	0.032*

Note: ^a^Steiger tests were conducted testing for differences between dependent correlations of R-GPTS scales with CAPE Paranoia to correlations between R-GPTS subscales and respective other scales. Reported correlations are Spearman’s rho. *P < 0.05, **P < 0.001

## Discussion

The goal in the present study was to assess the validity of the German version of the Revised-Green et al. Paranoid Thoughts Scale (R-GPTS) in non-clinical and clinical groups and to extend the psychometric evaluation of the two subscales to factor structure, measurement invariance, internal reliability, test-retest reliability, convergent and discriminant validity. Our findings demonstrated that the German R-GPTS aligns with the previously reported two-factor structure and the R-GPTS A subscale exhibited solid evidence in favour of metric invariance across sex and over time in the non-clinical group and between non-clinical and clinical groups. In contrast, there was only mixed evidence for measurement invariance models of the R-GPTS B subscale. Moreover, the R-GPTS presented with good-to-excellent internal consistency as well as good convergent and discriminant validity.

Our results align with the original validation study by Freeman and colleagues [13], which established the R-GPTS as a reliable and valid measure of paranoia in both clinical and non-clinical groups. While Freeman et al. [13] identified a clean two-factor structure with high reliability, our study extends these findings by demonstrating similar psychometric properties in a German-speaking sample. Additionally, we evaluated test-retest reliability and measurement invariance of the scale across sex, between clinical and non-clinical samples and between measurement time-points. Regarding test-retest reliability, the R-GPTS subscales showed fair-to-good reliability until 10-week follow-up except for the social reference subscale in the clinical group. Regarding measurement invariance, we found solid evidence in favour of metric invariance for the “Social Reference”-subscale across sex, over time (in the non-clinical group) and between clinical and non-clinical groups. This suggests the underlying construct is understood similarly between men and women, patients and healthy controls, and over time. At the same time, the evidence also suggested absence of scalar and residual invariance, so differences in scores may result from factors other than true variations in social reference. For the “Social Reference”-subscale, these results also align with findings by Kowalski *and colleagues* [14] who found evidence supporting metric invariance for the two R-GPTS factors using a Polish translation. Regarding the “Persecutory Ideations”-subscale, however, we observed more mixed evidence that only lent some support for measurement invariance across sex, patient status, and over time (based mostly on log-likelihood tests between models). Therefore, caution is warranted regarding score difference interpretations for this subscale in case-control and longitudinal settings and future work could consider scale modifications or alternative methods to improve measurement properties in diverse populations.

In addition to the psychometric extension of our R-GPTS validation, our study tested the scale’s performance in a psychiatric inpatient sample composed of individuals with PDD. This contributes new knowledge about the utility of the R-GPTS in assessing paranoia across different psychiatric conditions. Despite the exclusion of patients with acute psychosis from the PDD sample, we observed similar levels of R-GPTS subscale scores in PDD patients as compared to matched non-clinical individuals emphasising the continuous nature of paranoid thoughts beyond acute psychosis [11, 32, 33]. Future research should evaluate whether these milder levels of paranoia hold clinical relevance for prognosis and/ or treatment effectiveness in PDD.

During completion of the current study, another validation of a German version of the R-GPTS was published [34]. In this study, a German translation of the R-GPTS was evaluated in a representative sample of 516 participants from the German population regarding the cross-cultural measurement invariance of the two-factor solution. The authors could confirm scalar invariance, providing evidence in favour of the comparability of responses between English-speaking individuals and Germans, thereby supporting the notion that the underlying constructs measured by the questionnaire are understood similarly across these cultural groups. We expand upon this work by providing a comprehensive analysis that includes test-retest reliability over a 10-week period, concurrent and discriminant validity through established psychological measures such as the DASS-21 and the CAPE-P15, and criterion validity. Furthermore, we assessed measurement invariance across diverse groups, including clinical and non-clinical groups as well as across sexes. Taken together, the evidence from the representative German sample by Schlier and colleagues and our work provides a clear picture of the R-GPTS on the validity of the two-factor structure, test-retest reliability, concurrent and discriminant validity and metric invariance across cultures, time, and sex. At the same time, we need to acknowledge that the parallelism of the R-GPTS validation studies resulted in differences in the exact item translations to the German language, which we have summarised and discussed in more detail in the Supplement for the interested reader. Future research should investigate which combination of the two German translations provides a more accurate reflection of the underlying constructs, ultimately informing best practices in the assessment of paranoid thoughts.

There are multiple important strengths and limitations to the present work. First and as discussed above, one key strength of our study is the use of a well-defined clinical sample outside the context of acute psychosis, which allowed for a nuanced examination of the scale’s properties in a specific psychiatric context. However, this also represents a limitation, as we cannot report validation results for psychotic disorders or other mental disorders. Second, PDD patients in the current study were hospitalised for their depression. On the one hand, this biases our results towards the more severe end of PDD and, on the other hand, treatment effects from pharmaco- and/ or psychotherapy will have likely influenced our results on test-retest reliability and temporal invariance. Third, our test-retest reliability analyses focused on a 10-week interval rather than more common 1-week intervals [35], which reduces comparability to other studies and aggravates confounding effects (e.g., treatment in the clinical group) over the follow-up period. Fourth, descriptive analyses indicated floor effects for the “Persecutory Ideations”-subscale in that >50% of our samples indicated “0” across subscale items (cf. Figure 1). While mean and variance-adjusted weighted least squares estimation is suitable to deal with non-normality [36], the pronounced floor effects could have biased internal reliability and fit estimates. Additionally, previous work suggested higher mean values of the persecutory ideation subscale [10, 14, 34]; this also points to a potential limitation of the current translation of this subscale that needs to be evaluated in future work. Fifth, while our non-clinical sample was well-powered for CFA and measurement invariance analyses, sample size for the clinical sample was more restrictive and analyses exhibited larger variance. Sixth, our non-clinical sample was a convenience sample and is unrepresentative of the German general population posing the problem of selection bias. While we matched non-clinical and clinical samples based on age and sex, comparability in education was not achieved, thus contributing to potential bias in our results considering previously reported associations of paranoia with sociodemographic characteristics [8].

## Conclusion

In conclusion, the German version of the R-GPTS is a reliable and valid tool for assessing paranoia in both clinical and non-clinical groups. Our study corroborates the psychometric robustness of the scale as originally demonstrated by Freeman et al. (2021) and extends its applicability to a German-speaking population with persistent depression. Future research is required to address the identified gaps – particularly in measurement invariance for the “Persecutory Ideations”-subscale – and ensure the scale’s broader utility across diverse settings and populations.

## Supplementary Information

Below is the link to the electronic supplementary material.

Supplementary Material 1

## Data Availability

The datasets used and/or analysed during the current study are available from the corresponding author on reasonable request.

## Abbreviations

DASS-21: Depression, Anxiety and Stress Scales − 21 items
CAPE-P15: Community Assessment of Psychic Experiences - Positive Scale, 15 items
R-GPTS: Revised-Green et al. Paranoid Thoughts Scale
PDD: Persistent Depressive Disorder
CFI: Comparative Fit Index
TLI: Tucker–Lewis Index
RMSEA: Root Mean Square Error of Approximation
SRMR: Standardized Root Mean Square Residual
ICC: Intra-class Correlation Coefficient
α: Cronbach’s Alpha
