# Supplementary material for: A psychometric evaluation of the German Revised-Green et al. Paranoid Thoughts Scale (R-GPTS) in clinical and non-clinical groups
Source: BMC Psychiatry. 2025 Nov 18;25:1095. doi: 10.1186/s12888-025-07538-0 (PMC12625113; doi:10.1186/s12888-025-07538-0)
Supplement: Supplementary file 1 — Supplementary Material 1 [file 12888_2025_7538_MOESM1_ESM.docx]

**Online Supplement**

A psychometric evaluation of the German Revised-Green et al. Paranoid Thoughts Scale (R-GPTS) in clinical and non-clinical groups

**Contents**

[Table 1. R-GPTS Item descriptives 2](#_Toc202255597)

[Figure 1. R-GPTS item intercorrelations 3](#_Toc202255598)

[Table 2. R-GPTS factor loadings and item-factor correlations 4](#_Toc202255599)

[Table 3. Test-retest reliability 6](#_Toc202255600)

[Table 4. Comparison of the different translations of the R-GPTS 7](#_Toc202255601)

[Supplementary Summary of Translations and Degree of Similarity. 9](#_Toc202255602)

[Supplementary Discussion 12](#_Toc202255603)

# Table 1. R-GPTS Item descriptives

|  |  |  | Likert response (%) | | | | |
| --- | --- | --- | --- | --- | --- | --- | --- |
| Item | Mean | SD | 0 | 1 | 2 | 3 | 4 |
| A1 | 0.79 | 1.16 | 59 | 19 | 10 | 7 | 4 |
| A2 | 0.71 | 0.97 | 57 | 24 | 11 | 8 | 0 |
| A3 | 1.06 | 1.27 | 48 | 21 | 14 | 11 | 6 |
| A4 | 0.74 | 1.09 | 61 | 17 | 13 | 6 | 3 |
| A5 | 0.89 | 1.24 | 56 | 19 | 9 | 10 | 5 |
| A6 | 0.75 | 1.09 | 62 | 14 | 15 | 8 | 2 |
| A7 | 1.43 | 1.38 | 36 | 21 | 18 | 14 | 11 |
| A8 | 0.94 | 1.32 | 58 | 15 | 10 | 9 | 8 |
| B1 | 0.43 | 0.94 | 78 | 11 | 5 | 4 | 2 |
| B2 | 0.19 | 0.66 | 90 | 4 | 2 | 2 | 1 |
| B3 | 0.58 | 1.05 | 70 | 15 | 7 | 5 | 3 |
| B4 | 0.14 | 0.56 | 92 | 4 | 2 | 1 | 1 |
| B5 | 0.39 | 0.92 | 80 | 10 | 5 | 3 | 3 |
| B6 | 0.22 | 0.69 | 88 | 7 | 3 | 2 | 1 |
| B7 | 0.12 | 0.51 | 93 | 4 | 1 | 1 | 0 |
| B8 | 0.31 | 0.87 | 85 | 6 | 3 | 2 | 3 |
| B9 | 0.44 | 0.96 | 77 | 11 | 5 | 4 | 3 |
| B10 | 0.49 | 1.02 | 77 | 7 | 9 | 4 | 3 |

# Figure 1. R-GPTS item intercorrelations


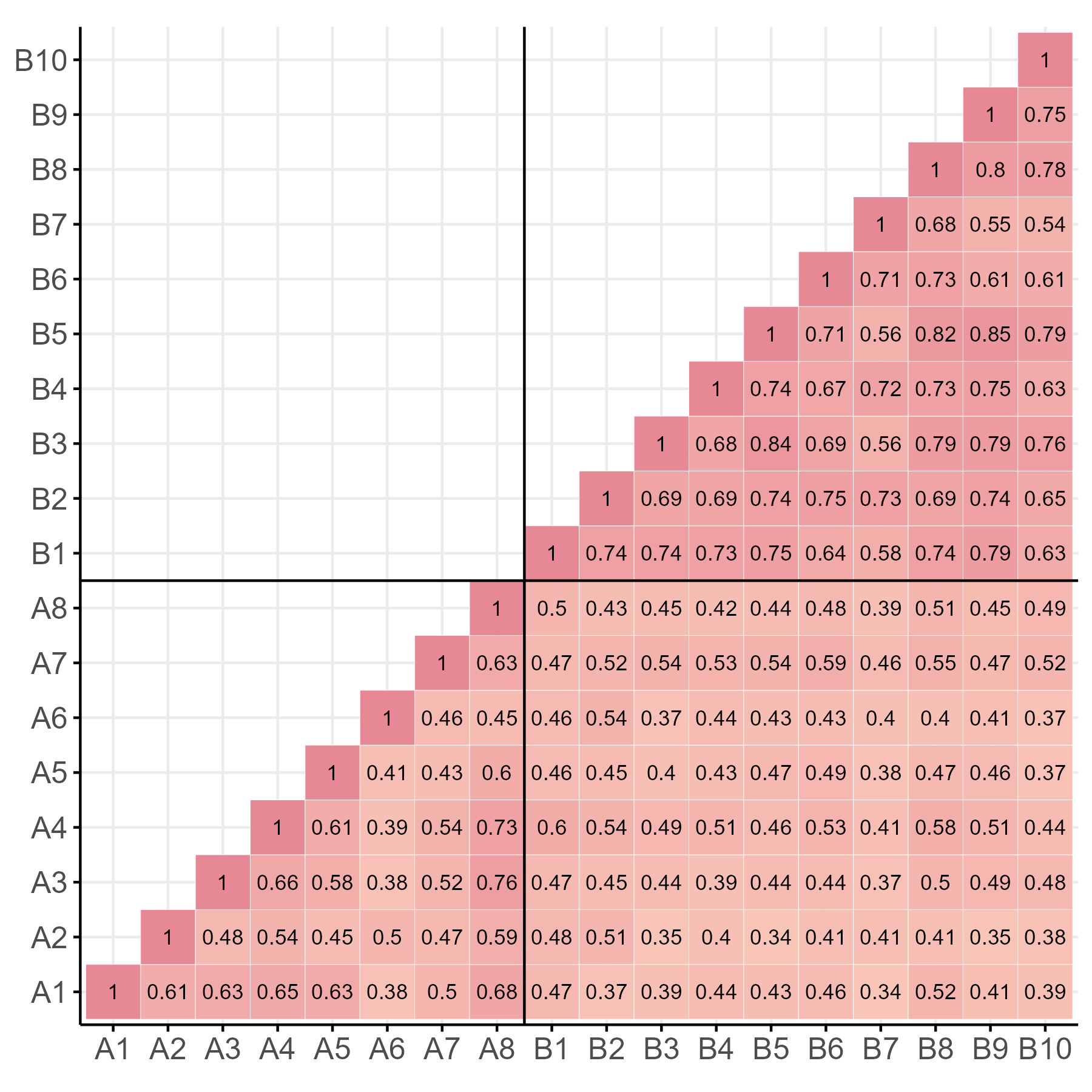


# Table 2. R-GPTS factor loadings and item-factor correlations

|  |  | Spearman’s rho | | Correlation difference  P-values^c^ |
| --- | --- | --- | --- | --- |
| Item | Factor loading^a^ | R-GPTS A | R-GPTS B |  |
| A1 | 0.80 | 0.71 | 0.38 | <0.001** |
| A2 | 0.69 | 0.60 | 0.36 | <0.001** |
| A3 | 0.81 | 0.79 | 0.39 | <0.001** |
| A4 | 0.81 | 0.72 | 0.41 | <0.001** |
| A5 | 0.71 | 0.64 | 0.39 | <0.001** |
| A6 | 0.55 | 0.48 | 0.36 | <0.001** |
| A7 | 0.67 | 0.68 | 0.46 | <0.001** |
| A8 | 0.90 | 0.84 | 0.40 | <0.001** |
| B1 | 0.84 | 0.42 | 0.67 | <0.001** |
| B2 | 0.84 | 0.32 | 0.49 | <0.001** |
| B3 | 0.89 | 0.42 | 0.81 | <0.001** |
| B4 | 0.83 | 0.27 | 0.45 | <0.001** |
| B5 | 0.93 | 0.38 | 0.72 | <0.001** |
| B6 | 0.79 | 0.34 | 0.52 | <0.001** |
| B7 | 0.74 | 0.24 | 0.38 | <0.001** |
| B8 | 0.90 | 0.38 | 0.62 | <0.001** |
| B9 | 0.90 | 0.40 | 0.74 | <0.001** |
| B10 | 0.83 | 0.38 | 0.69 | <0.001** |

Note: ^a^Factor loadings correspond to CFA loadings with the associated factor (i.e., A1 with R-GPTS A, A2 with R-GPTS A, B1 with R-GPTS B, B2 with R-GPTS B, etc.). ^b^Spearman’s rho correlations were calculated between individual R-GPTS items and latent factor estimates from CFA for R-GPTS A and R-GPTS B. This approach was chosen to examining item associations with the respective other scale rather than cross-loadings, which would have required a joint CFA approach. ^c^Steiger tests were conducted testing for differences between dependent correlations of R-GPTS items with R-GPTS A and B subscale factor scores. *P<0.05, **P<0.001

# Table 3. Test-retest reliability

|  | Non-clinical sample | |  | Patients | |
| --- | --- | --- | --- | --- | --- |
| R-GPTS Scale | Spearman’s rho | ICC |  | Spearman’s rho | ICC |
| A: Social Reference | 0.59 | 0.62 |  | 0.51 | 0.34 |
| B: Persecutory Ideations | 0.56 | 0.59 |  | 0.63 | 0.48 |

# Table 4. Comparison of the different translations of the R-GPTS

|  | Version  Schlier et al. (2024) | Version  Rek et al. (in prep.) | Translation similarity* |
| --- | --- | --- | --- |
| **Part A** |  |  |  |
| Item 1 | Ich habe Zeit damit verbracht darüber nachzudenken, ob Freunde über mich lästern. | Ich habe länger darüber nachgedacht, wie Freunde Tratsch und Klatsch über mich verbreitet haben. | Moderate |
| Item 2 | Ich habe oft mitbekommen, dass Menschen über mich geredet haben. | Ich habe oft gehört, wie Leute mich erwähnt haben. | Moderate |
| Item 3 | Es hat mich belastet, wenn Freunde und Kollegen mich kritisiert haben. | Es hat mich verletzt, dass Freunde oder Kollegen mich kritisch beurteilten. | Moderate |
| Item 4 | Menschen haben mit Sicherheit hinter meinem Rücken über mich gelacht. | Menschen haben sicher hinter meinem Rücken über mich gelacht. | High |
| Item 5 | Ich habe viel darüber nachgedacht, ob Menschen den Kontakt zu mir vermeiden | Ich habe viel darüber nachgedacht, dass andere Menschen mich meiden. | Moderate |
| Item 6 | Menschen haben mir gegenüber Andeutungen gemacht. | Menschen haben mir Hinweise gegeben. | Moderate |
| Item 7 | Ich glaubte, dass bestimmte Leute nicht diejenigen waren, als die sie erschienen. | Ich habe geglaubt, dass bestimmte Menschen nicht so sind, wie sie zu sein scheinen. | Moderate |
| Item 8 | Dass Menschen hinter meinem Rücken über mich reden, hat mich belastet. | Dass Menschen hinter meinem Rücken über mich geredet haben, hat mich verletzt. | Moderate |
| **Part B** |  |  |  |
| Item 1 | Bestimmte Personen hatten es auf mich abgesehen. | Bestimmte Personen hatten es auf mich abgesehen. | High |
| Item 2 | Menschen starrten mich an, damit ich mich bedroht fühlte. | Menschen wollten, dass ich mich bedroht fühle, also starrten sie mich an. | Moderate |
| Item 3 | Ich war mir sicher, dass bestimmte Personen Dinge getan haben, um mich zu nerven. | Ich war mir sicher, dass Menschen Dinge getan haben, um mich zu verärgern. | Moderate |
| Item 4 | Ich war davon überzeugt, dass es eine Verschwörung gegen mich gab. | Ich war überzeugt, dass es eine Verschwörung gegen mich gab. | High |
| Item 5 | Ich war mir sicher, dass mir jemand Leid zufügen wollte. | Ich war mir sicher, dass jemand mich verletzen wollte. | Moderate |
| Item 6 | Ich konnte nicht aufhören, daran zu denken, dass Menschen mich verwirren wollen. | Ich konnte nicht aufhören, zu denken, dass andere Menschen mich verwirren wollten. | Moderate |
| Item 7 | Es hat mich belastet, verfolgt zu werden. | Ich war beunruhigt, weil ich verfolgt wurde. | Moderate |
| Item 8 | Es war schwierig, nicht daran zu denken, dass Menschen wollten, dass es mir schlecht geht. | Es war schwierig, nicht mehr daran zu denken, dass andere Menschen wollten, dass ich mich schlecht fühle. | Moderate |
| Item 9 | Menschen waren mir gegenüber absichtlich feindselig. | Menschen haben sich mir gegenüber mit Absicht feindselig verhalten. | High |
| Item 10 | Ich war wütend darüber, dass mir jemand Leid zufügen wollte. | Ich war wütend, dass mich jemand verletzen wollte. | Moderate |

* For a detailed discussion on similarities between these translations, please see the subsequent sections.

# Supplementary Summary of Translations and Degree of Similarity.

The two versions of the questionnaire items from Schlier et al. (2024) and the current publication display some noteworthy differences in phrasing and emphasis, which could have influenced the interpretation and clarity of the items. Below is a summary of the differences for each part, along with a brief discussion of their potential implications for scientific research.

**Part A:**

1. **Item 1**: *I spent time thinking about friends gossiping about me.*
   - **Schlier et al.**: "Ich habe Zeit damit verbracht darüber nachzudenken, ob Freunde über mich lästern."
   - **Rek et al.**: "Ich habe länger darüber nachgedacht, wie Freunde Tratsch und Klatsch über mich verbreitet haben."
   - **Similarity**: Moderate. Schlier et al. focuses on thoughts about gossip, while Rek et al. implies a more active role of friends.
   - **Our conclusion**: **Schlier et al.** captures the essence of the original better.
2. **Item 2**: *I often heard people referring to me.*
   - **Schlier et al.**: "Ich habe oft mitbekommen, dass Menschen über mich geredet haben."
   - **Rek et al.**: "Ich habe oft gehört, wie Leute mich erwähnt haben."
   - **Similarity**: Moderate. Schlier et al. implies discussions, while Rek et al. emphasizes mere mentions.
   - **Our conclusion**: **Rek et al.** is slightly closer as it captures the passive nature of "referring to."
3. **Item 3**: *I have been upset by friends and colleagues judging me critically.*
   - **Schlier et al.**: "Es hat mich belastet, wenn Freunde und Kollegen mich kritisiert haben."
   - **Rek et al.**: "Es hat mich verletzt, dass Freunde oder Kollegen mich kritisch beurteilten."
   - **Similarity**: Moderate. Both convey an emotional response, but Rek et al. emphasizes personal hurt more explicitly.
   - **Our conclusion**: **Rek et al.** as it aligns more closely with the original expression of distress.
4. **Item 4**: *People definitely laughed at me behind my back.*
   - **Schlier et al.**: "Dass Menschen hinter meinem Rücken über mich gelacht."
   - **Rek et al.**: "Menschen haben sicher hinter meinem Rücken über mich gelacht."
   - **Similarity**: High. Both translations effectively communicate the same idea with minor variations.
   - **Our conclusion**: **Both translations** are equally apt.
5. **Item 5**: *I have been thinking a lot about people avoiding me.*
   - **Schlier et al.**: "Ich habe viel darüber nachgedacht, ob Menschen den Kontakt zu mir vermeiden."
   - **Rek et al.**: "Ich habe viel darüber nachgedacht, dass andere Menschen mich meiden."
   - **Similarity**: Moderate. Schlier et al. introduces uncertainty, while Rek et al. is more direct.
   - **Our conclusion**: **Rek et al.**, for its directness in expressing the thought of avoidance.
6. **Item 6**: *People have been dropping hints for me.*
   - **Schlier et al.**: "Menschen haben mir gegenüber Andeutungen gemacht."
   - **Rek et al.**: "Menschen haben mir Hinweise gegeben."
   - **Similarity**: Moderate. "Andeutungen" implies ambiguity; "Hinweise" is clearer.
   - **Our conclusion**: **Rek et al.** as it conveys a clearer understanding of hints.
7. **Item 7**: *I believed that certain people were not what they seemed.*
   - **Schlier et al.**: "Ich glaubte, dass bestimmte Leute nicht diejenigen waren, als die sie erschienen."
   - **Rek et al.**: "Ich habe geglaubt, dass bestimmte Menschen nicht so sind, wie sie zu sein scheinen."
   - **Similarity**: Moderate. Both capture the essence but differ in phrasing and tense.
   - **Our conclusion**: **Rek et al.** as it maintains a more present relevance, similar to the original.
8. **Item 8**: *People talking about me behind my back upset me.*
   - **Schlier et al.**: "Dass Menschen hinter meinem Rücken über mich reden, hat mich belastet."
   - **Rek et al.**: "Dass Menschen hinter meinem Rücken über mich geredet haben, hat mich verletzt."
   - **Similarity**: Moderate. Schlier et al. suggests a burden, while Rek et al. emphasizes emotional pain.
   - **Our conclusion**: **Rek et al.** as it mirrors the emotional impact more closely.

**Part B:**

1. **Item 1**: *Certain individuals have had it in for me.*
   - **Schlier et al.**: "Bestimmte Personen hatten es auf mich abgesehen."
   - **Rek et al.**: "Bestimmte Personen hatten es auf mich abgesehen."
   - **Similarity**: High. Both are identical and accurately reflect the original item.
   - **Our conclusion**: **Both translations** are equally suitable.
2. **Item 2**: *People wanted me to feel threatened, so they stared at me.*
   - **Schlier et al.**: "Menschen starrten mich an, damit ich mich bedroht fühlte."
   - **Rek et al.**: "Menschen wollten, dass ich mich bedroht fühle, also starrten sie mich an."
   - **Similarity**: Moderate. Schlier et al. implies a direct causation, while Rek et al. emphasizes the intention more clearly.
   - **Our conclusion**: **Rek et al.** for its clarity regarding the intent behind the staring.
3. **Item 3**: *I was certain people did things in order to annoy me.*
   - **Schlier et al.**: "Ich war mir sicher, dass bestimmte Personen Dinge getan haben, um mich zu nerven."
   - **Rek et al.**: "Ich war mir sicher, dass Menschen Dinge getan haben, um mich zu verärgern."
   - **Similarity**: Moderate. Both convey a sense of certainty. Rek et al. uses "upset" instead of "annoy."
   - **Our conclusion**: **Schlier et al.** as the focus on "annoy" may align better with the original's context.
4. **Item 4**: *I was convinced there was a conspiracy against me.*
   - **Schlier et al.**: "Ich war davon überzeugt, dass es eine Verschwörung gegen mich gab."
   - **Rek et al.**: "Ich war überzeugt, dass es eine Verschwörung gegen mich gab."
   - **Similarity**: High. Both share the same meaning and structure.
   - **Our conclusion**: **Both translations** effectively convey the original intent.
5. **Item 5**: *I was sure someone wanted to hurt me.*
   - **Schlier et al.**: "Ich war mir sicher, dass mir jemand Leid zufügen wollte."
   - **Rek et al.**: "Ich war mir sicher, dass jemand mich verletzen wollte."
   - **Similarity**: Moderate. Rek et al.'s translation uses "hurt," which is more direct than "cause harm."
   - **Our conclusion**: **Rek et al.** as it mirrors the original phrasing more closely.
6. **Item 6**: *I couldn’t stop thinking about people wanting to confuse me.*
   - **Schlier et al.**: "Ich konnte nicht aufhören, daran zu denken, dass Menschen mich verwirren wollen."
   - **Rek et al.**: "Ich konnte nicht aufhören, zu denken, dass andere Menschen mich verwirren wollten."
   - **Similarity**: Moderate. Both capture the idea, but Rek et al. uses "other people" for clarity.
   - **Our conclusion**: **Rek et al.** for its clarity and past tense consistency.
7. **Item 7**: *I was distressed by being persecuted.*
   - **Schlier et al.**: "Es hat mich belastet, verfolgt zu werden."
   - **Rek et al.**: "Ich war beunruhigt, weil ich verfolgt wurde."
   - **Similarity**: Moderate. The emotional response differs: "burdened" vs. "worried."
   - **Our conclusion**: **Rek et al.** as it accurately reflects personal emotional distress.
8. **Item 8**: *It was difficult to stop thinking about people wanting to make me feel bad.*
   - **Schlier et al.**: "Es war schwierig, nicht daran zu denken, dass Menschen wollten, dass es mir schlecht geht."
   - **Rek et al.**: "Es war schwierig, nicht mehr daran zu denken, dass andere Menschen wollten, dass ich mich schlecht fühle."
   - **Similarity**: Moderate. Rek et al. adds "not to think anymore," enhancing clarity.
   - **Our conclusion**: **Rek et al.** for its clearer representation of the difficulty in stopping negative thoughts.
9. **Item 9**: *People have been hostile towards me on purpose.*
   - **Schlier et al.**: "Menschen waren mir gegenüber absichtlich feindselig."
   - **Rek et al.**: "Menschen haben sich mir gegenüber mit Absicht feindlich verhalten."
   - **Similarity**: High. Both effectively communicate intentional hostility.
   - **Our conclusion**: **Both translations** accurately convey the original intent.
10. **Item 10**: *I was angry that someone wanted to hurt me.*
    - **Schlier et al.**: "Ich war wütend darüber, dass mir jemand Leid zufügen wollte."
    - **Rek et al.**: "Ich war wütend, dass mich jemand verletzen wollte."
    - **Similarity**: Moderate. Rek et al. maintains direct phrasing with "hurt."
    - **Our conclusion**: **Rek et al.** as it stays true to the original wording.

# Supplementary Discussion

The differences in phrasing between Schlier et al. and our translation can significantly influence how respondents interpret the items. For instance, the more precise language in our translations—such as in Item 1 (Part A), where it states “friends have spread gossip” compared to the more idiomatic “friends gossip” used by Schlier et al.—might encourage participants to think more about the agency of others in their social interactions. This distinction could have implications for how concepts of *ideas of reference* are assessed in psychological research.

Furthermore, the greater emphasis on emotional pain in our translation might prompt stronger emotional reactions from participants, potentially leading to different response patterns and altering the understanding of the instrument's impact on them. These variations highlight the complexities involved in translating psychological measures. Even subtle changes in wording can significantly alter the intended meaning, making careful consideration essential to maintain the reliability and validity of the scale across different language versions.

Therefore, future research should explore how these nuances affect the psychometric properties of the instruments, ensuring they accurately capture the constructs they are designed to measure. It is also crucial to strike a balance between fidelity to the original phrasing and the need for clarity and relatability, as seen in Schlier et al.'s more idiomatic approach, which may enhance understanding for German respondents. Understanding these dynamics is key to effectively and meaningfully deploy psychological assessments in diverse cultural contexts.
